# Methylphenidate for the cognitive and neurobehavioural sequelae of traumatic brain injury in adults: a systematic review and meta-analysis

**DOI:** 10.3389/fneur.2025.1546080

**Published:** 2025-03-05

**Authors:** Jemima L. C. Rees, Rachel Saunders, Carl R. Krynicki, Antonio Belli, Zubair Ahmed, Valentina Di Pietro, Andrew R. Stevens

**Affiliations:** ^1^Neuroscience and Ophthalmology, Department of Inflammation and Ageing, School of Infection, Inflammation and Immunology, College of Medicine and Health, University of Birmingham, Birmingham, United Kingdom; ^2^School of Psychology, University of Birmingham, Birmingham, United Kingdom; ^3^Department of Clinical Neuropsychology, Queen Elizabeth Hospital Birmingham, Birmingham, United Kingdom; ^4^Department of Neurosurgery, Queen Elizabeth Hospital Birmingham, Birmingham, United Kingdom; ^5^University Hospitals Birmingham NHS Foundation Trust, Birmingham, United Kingdom

**Keywords:** methylphenidate, cognition, depression, fatigue, traumatic brain injury

## Abstract

**Introduction:**

Traumatic brain injury (TBI) is a leading cause of death and disability globally and is associated with long-term cognitive and neurobehavioural deficits. Methylphenidate has been proposed to address these lasting symptoms, however comprehensive evidence is lacking.

**Methods:**

This systematic review aimed to assess the effects of methylphenidate on multiple cognitive and neurobehavioural domains in adults with TBI. The search conducted across five databases yielded 1,019 results, of which 25 were relevant to this review. Meta-analyses were conducted where homogenous data was available.

**Results:**

Significant results favouring methylphenidate were recorded by meta-analyses for one of five cognition outcome measures (Trail Making Test A) (*p* = 0.005, CI [−5.19, −0.91]), as well as the depression domain (*p* < 0.00001, CI [−0.78, −0.39]) and the fatigue domain (*p* < 0.00001, CI [−0.98, −0.67]). Insufficient data was available in the aggression, apathy, agitation, memory, motor function, post-concussion syndrome and sleep domains for inclusion in meta-analysis. Qualitative review of evidence in these domains found limited and mixed evidence on the efficacy of methylphenidate, though significant benefits have been demonstrated in these various domains in small, randomised studies. Eleven of the 25 studies were judged as containing some to high risk of bias. However, this review identified supportive evidence for the beneficial effects of methylphenidate to improve depression and fatigue in adults with TBI, with some possible benefits for cognition and other symptoms. Heterogeneity was high and risk of bias was variable across studies, somewhat limiting credibility of results.

**Discussion:**

Methylphenidate may enhance the ongoing care of TBI patients, by addressing neurobehavioural and cognitive symptoms simultaneously. Further large-scale and high-quality clinical trials evaluating a comprehensive range of possible benefits to symptoms should be conducted to more conclusively elucidate the potential of methylphenidate for clinical efficacy in TBI.

## 1 Introduction

Traumatic brain injury (TBI) is a principal cause of death and disability globally, with approximately 69 million incidents occurring worldwide each year (1). Road traffic collisions, assaults and falls are the primary causes, with young people and the elderly being disproportionately affected (1). Individuals who suffer moderate to severe TBI are significantly more likely to experience long-term psychological, behavioural and cognitive deficits than those with less severe injury, imposing substantial personal and financial burden on patients, caregivers and health systems (2, 3).

Management of persistent TBI symptoms is multidisciplinary, consisting of physical, cognitive and psychological rehabilitation (4).^1^ Effective treatment of long-term TBI sequelae is limited by poor understanding of the heterogenous pathology and the range of cognitive and neurobehavioural domains that are affected (2). Consequently, pharmacological intervention in the post-acute and chronic stages of TBI often results in polypharmacy, with some patients being prescribed up to six or more drugs simultaneously (5).

Methylphenidate (MPh) is licenced for use in the UK to treat both attention deficit hyperactivity disorder (ADHD) and narcolepsy (6),^2^ conditions that share features with persistent symptoms of TBI. Available under various brand names in both immediate-release and long-acting preparations, MPh acts on the noradrenergic and dopaminergic systems by blocking reuptake of neurotransmitters noradrenaline and dopamine, thereby exerting a stimulant effect (7). As such, the potential of MPh in addressing cognitive deficits following TBI has been a subject of growing interest over the last 40 years (8–10).

The rationale for this lies in its mechanism of action, which theoretically may enhance cognitive domains commonly affected by TBI, such as processing speed, executive function, and attention (7). More recently, research has been expanded to investigate the effects of MPh on fatigue, memory and motor function in TBI (11–13). Despite these advances, there is currently no systematic review that comprehensively documents all available evidence on the effects of MPh on the long-term cognitive and neurobehavioural consequences of TBI. The primary aim of this systematic review is to synthesise evidence regarding the efficacy of MPh in treating common sequelae of TBI in adults, specifically, aggression, agitation, apathy, cognition, depression, fatigue, memory, motor function, post-concussion syndrome (PCS) and sleep disturbance.

## 2 Materials and methods

### 2.1 Study protocol

This report adheres to the Preferred Reporting Items for Systematic Review and Meta-Analyses (PRISMA) guidelines (14) and was guided by the Cochrane Handbook for Systematic Reviews of Interventions (15).

### 2.2 Search strategy

The search terms outlined in Table 1 were applied to five bibliographic databases (PubMed, EMBASE, PsycInfo, Ovid and CINAHL) on 11th June 2024. No publication date restrictions were applied to the search. The relevant syntax was applied for each database.

**Table 1:** Search strategy.

Search terms	Inclusion criteria	Exclusion criteria
“traumatic brain injury” OR “brain injury” OR “head injury” OR “TBI” AND “methylphenidate” OR “Ritalin” OR “Concerta” OR “central nervous system stimulant” AND “aggression” OR “agitation” OR “apathy” or “cognition” OR “cognitive” OR “executive function” OR “memory” OR “depression” OR “depressive symptoms” OR “mood” OR “fatigue” OR “tiredness” OR “irritability” OR “sleep disturbances” OR “sleep disorders” OR “insomnia” OR “post-concussion syndrome” OR “motor” AND “randomised controlled trial” OR “clinical trial” OR “cohort study” OR “case–control” OR non-randomised” OR “observational study”	Adults >18 years with TBI diagnosis Administration of methylphenidate Studies published in or translated to English Study types: - RCTs (including crossover and parallel designs) - Non-randomised control trials - Observational studies - Clinical trials with control groups One or more of the following outcomes reported: aggression, agitation, apathy, cognition, memory, motor function, depression, fatigue, sleep disturbances	Paediatric sample (<18 years) Acute phase of injury (≤72 h post-injury) Studies not available in English Study types: - Systematic reviews - Narrative reviews - Case reports - Editorials - Opinion pieces - Observational studies without a control group

During pilot searches, the majority of studies described enrolment of participants with TBI >72 h post-injury, with very few studies describing instigation of MPh treatment within the acute (<72 h post-injury) phase. In an attempt to mitigate heterogeneity of inclusion of both the acute and sub-acute/chronic phases, these few studies describing acute enrolment were excluded. Similarly, clinical studies enrolling patients with TBI typically results in greater heterogeneity owing to acute uncertainty in prognosis. Early enrolment requires classification of TBI to be reliant on presenting GCS: due to varying confounding factors this is less accurate a measure compared to assessment after 72 h where clinical assessment (e.g., of post-traumatic amnesia) allows for more accurate clinical classification, as well as assessment of baseline symptoms.

Furthermore, pharmacological intervention with interference in neurotransmitter pathways may offer some neuroprotective effects. Whilst this may be a mechanism of MPh, such effects were considered out of scope of this systematic review which aimed to comprehensively capture studies which employed MPh as a later intervention to promote symptomatic recovery. The differences in acute and sub-acute/chronic processes after neurotrauma have been well described in extensive reviews which we have previously published elsewhere (16). Due to these factors in combination, it was determined by the authors that the benefits of inclusion of these acute studies offered greater confounders and could likely lead to less clarity in the conclusions than were they excluded.

Two independent reviewers (JR and RS) then undertook the full screening process from searches in the databases. Discrepancies were discussed with a third party (ARS) after which consensus was reached. Duplicates were removed using EndNote duplicate detection software (17) which was double-checked by hand. Following title and abstract screening, full text screening was completed using the exclusion criteria outlined in Table 1. Further records were identified from citation screening of included studies and relevant systematic reviews. Studies assessing the effect of MPh during the acute phase (<72 h post-TBI) were excluded due to the distinct pathophysiological changes characteristic of this stage (18).

### 2.3 Data extraction

Data extraction was performed independently by two reviewers (JR and RS). A pre-defined, piloted data extraction form, developed based on the Cochrane Handbook (15),^3^ was used to ensure consistency between reviewers, and the *a priori* methodology was for any discrepancies to be resolved via discussion with a third, independent reviewer. The following information was compiled from each included study: (i) study characteristics: first author, publication year, country, study design; (ii) participant characteristics: sample size, mean age, gender, mean time since TBI; (iii) intervention details: frequency, dose, duration; and (iv) outcome measures with results. In the event of missing or inadequate data reporting, the corresponding author was contacted to request access to the primary data. In the conduct of this systematic review, no discrepancies between reviewers in study inclusion nor extracted data points were identified, with 100% concordance between the primary reviewers and hence inter-rater reliability was not formally assessed.

### 2.4 Risk of bias analysis

Risk of bias analysis was performed independently, in duplicate, by two authors (JR and RS). The RoB2 tool was used for parallel and crossover randomised controlled trials (RCT) (19); ^4^the SCED Scale was used to evaluate single-case experimental design studies (20); and the ROBINS-1 tool was used for observational studies (21). Discrepancies were settled via discussion with a third author (ARS), after which consensus was reached. In the case of discrepancies, we had also planned for further authors to provide critical appraisal of the studies followed by open discussion and voting. However, no discrepancies in the risk of bias were identified, and hence such steps to achieve consensus were not required.

### 2.5 Statistical analysis

RevMan version 5.4.1 (22) was used to perform meta-analyses of outcome measures reported by three or more studies recording MPh versus placebo. Data input was overseen by two reviewers (JR and ARS). Studies were excluded from meta-analysis where it was not possible to ascertain the distribution of results due to missing or unavailable data. Meta-analysis was conducted where three or more studies used the same outcome measure, and therefore only five outcome measures permitted meta-analysis. Due to heterogenous data reporting, effect size was calculated with a 95% confidence interval using standardised mean difference model. Where heterogeneity was <50% (Chi^2^ test), a fixed effects model was used; where ≥50%, a random effects model was used. Where no overall score was available for an outcome measure, a pooled mean difference and standard error was calculated. For continuous outcomes such as depression and fatigue that were reported using a range of outcome measures, standardised mean difference, using Cohen’s *d*, was calculated (23). This method is flawed it its inability to account for heterogeneity between study samples and reliability of each outcome measure, however its use when comparing outcome measures of continuous variables has been justified (23).

## 3 Results

### 3.1 Study selection

**Figure 1:**
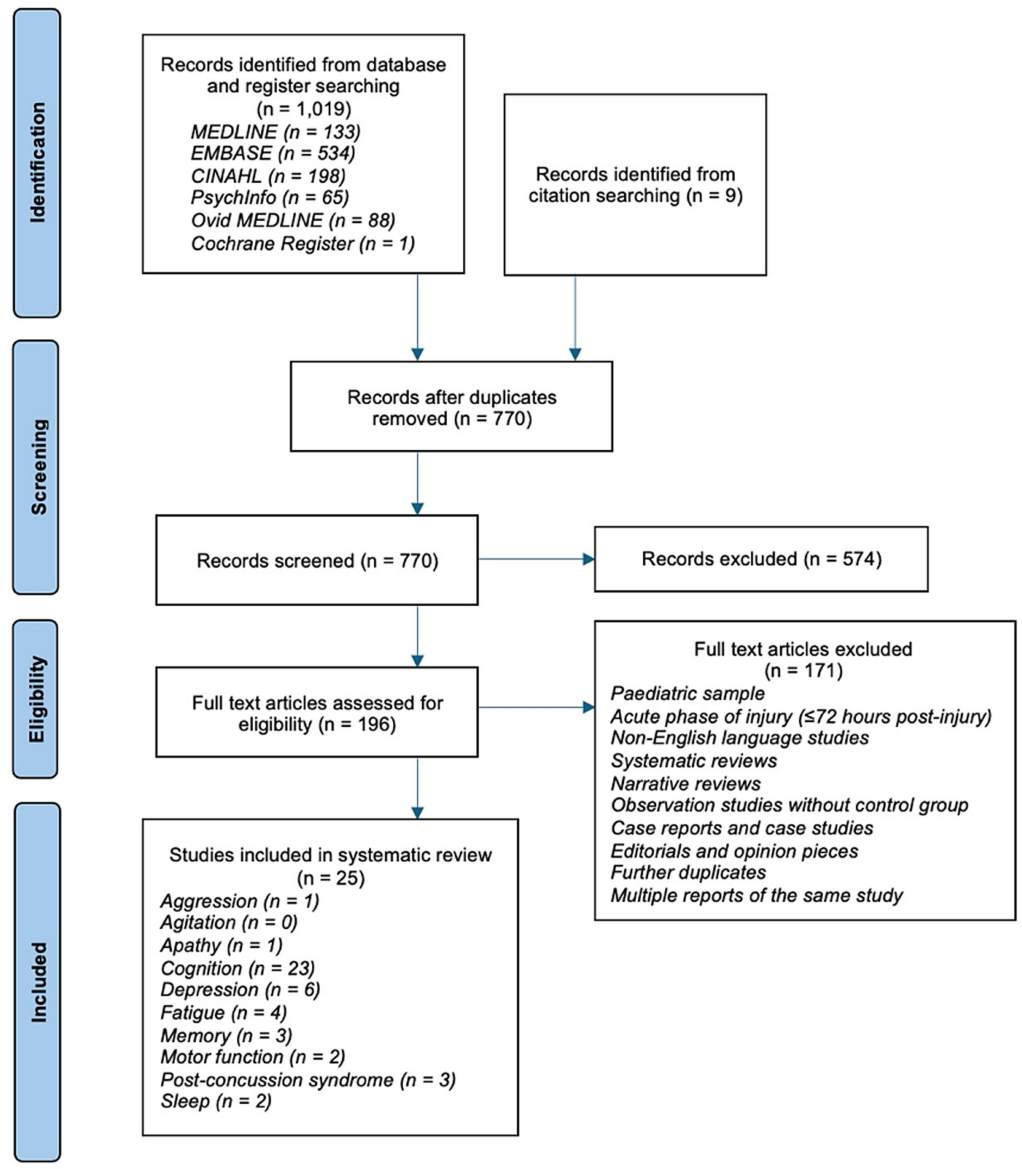
PRISMA flow diagram (14). Some studies reported outcomes across multiple domains, hence the total number of included studies does not match the sum of reported domains.

The search results are outlined in a PRISMA flow diagram shown in Figure 1. There were 1,019 records identified, with 770 remaining following removal of duplicates. Title and abstract screening identified 196 studies from which full text eligibility screening identified 25 studies (8–13, 24–45). Records reporting on the same patient group were considered the same study for the purpose of this review; three studies were identified to report a single group of patients over longitudinal follow-up (24–26).

### 3.2 Characteristics of the included studies

This review assessed the existing literature on the effect of MPh on post-TBI aggression, agitation, apathy, cognition, depression, fatigue, memory, motor function, post-concussion syndrome and sleep. The included studies comprised 22 RCTs (8–13, 29–31, 33–45), two single-case experimental designs (SCED) (27, 32) and one observational study with a control cohort (27). Detailed study characteristics are provided in Table 2. Thirteen studies were from the US (8, 9, 27, 28, 32, 33, 36–42, 44), four from the UK (13, 30, 35, 40), two from Sweden (11, 31), two from Australia (29, 45), two from South Korea (12, 34), one from China (10) and one from Argentina (43). A total of 666 individuals participated in the included studies with a mean age of 36.18 years. The proportion of males was 69.41% with TBI severity ranging from mild to severe. Time since TBI until enrolment on the study ranged from 4 days to 8.6 years whilst the dose of MPh treatment ranged from 5 mg od (once daily) to 90 mg od; duration of treatment ranged from one dose to 30 weeks.

**Table 2:** Detailed characteristics of included studies.

Study ID	Country	Design	Total n	Age, mean (±SD) [range]	Male (%)	TBI severity^*^	Time since TBI, mean (±SD) [range]	MPh dose	MPh duration	Comparator	Outcome domains
Al-Adawi et al. (27)	USA	Single case experimental design (AAB)	10	38 (17.39)	90	Mild to moderate	63.8 days	5–10 mg od	2 weeks	Baseline tests x 2	Cognition
Al-Adawi et al. (28)	USA	Prospective cohort	30	51 [18–88]	76.67	NI	NI	5–10 mg od	Variable	13 patients not taking MPh	Cognition Sleep
Dorer et al. (13)	UK	RCT crossover	14	37.27 (13.38)	78.57	Mild to severe	23.43 months	30 mg	0–2 doses (depending on randomisation)	Placebo	Motor function
Dymowski et al. (29)	Australia	RCT parallel	11	34 (14.62)	66.67	Severe	293 days (104.76)	Up to 60 mg od	8 weeks	Placebo	Cognition
Gualtieri et al. (8)	USA	RCT crossover	15	24.1 (9.41)	66.67	Severe	46.8 months (41.3)	0.3 mg/kg bd or 0.15 mg/kg bd	4 weeks (2 weeks per condition)	Placebo	Cognition
Jenkins et al. (30)	UK	RCT crossover	40	Normal caudate DaT: 40 (11) Low caudate DaT: 39 (12)	Normal caudate DaT: 77 Low caudate DaT: 94	Moderate to severe	Normal caudate DaT: 67 months (86) Low caudate DaT: 86 months (93)	Up to 25 mg od	2 weeks	Placebo	Apathy Cognition Depression Fatigue
Johansson et al. (31)	Sweden	RCT crossover	24	38.6 (11.1)	50	Mild to moderate	8.6 years (5.1)	5–15 mg od or 20–60 mg od	8 weeks (4 weeks per condition)	Placebo	Depression Fatigue
Johansson et al. (11)	Sweden	RCT crossover	44	38.9 (10.8)	43.18	Mild to moderate	8.2 years (5.7)	5–15 mg od or 20–60 mg od	8 weeks (4 weeks per condition)	Placebo	Cognition Depression Fatigue
Johansson et al. (24)	Sweden	RCT crossover (6-month follow-up)	30	39.7 (12.5)	30	Mild to moderate	8.6 years (5.9)	15–90 mg od	6 months	Baseline	Cognition Depression Fatigue
Johansson et al. (25)	Sweden	RCT crossover (5.5 years follow-up)	17	46 (9)	41.18	Mild	NI	NI	5.5 years	Baseline	Cognition Depression Fatigue
Kaelin et al. (32)	USA	Single case experimental design (AABA)	11	51.5 [25–82]	72.73	Mild to severe	19.8 days (18.72)	Up to 15 mg od	9 days	Baseline tests x3	Cognition Depression
Kim et al. (33)	South Korea	RCT parallel	18	34.2 (10.65)	88.89	NI	2.6 (1.95)	20 mg	Up to one dose	Placebo	Cognition
Kim et al. (34)	USA	RCT crossover	23	34.2 (11.5)	78.26	Moderate to severe	51.1 months (63.3)	0.3 mg/kg	Once	Placebo	Cognition
Lee et al. (12)	South Korea	RCT parallel	30	34.45 (10.15)	80	Mild to moderate	33.35 days (4.85)	Up to 20 mg od	4 weeks	Placebo	Cognition Depression Memory PCS Sleep
Manktelow et al. (35)	UK	RCT crossover	38	36 [23–49]	70.59	Mild to severe	20.4 months (11.82)	30 mg	Once	Placebo	Cognition
McAllister et al. (36)	USA	RCT parallel	32	41.19 (9.09)	54.14	Mild	NI	5–20 mg bd	12 weeks	Placebo	Cognition Depression PCS
McDonald et al. (37)	USA	RCT parallel	71	40.19 (13.49)	65.33	Mild to severe	7.56 (9.27)	Up to 30 mg bd	6 weeks	Placebo	Cognition
Mooney et al. (38)	USA	RCT parallel	38	29.45 (10.02)	100	Severe	27.08 months (21.13)	Up to 30 mg od	6 weeks	Baseline	Aggression Cognition
Newsome et al. (39)	USA	RCT parallel	9	39.19 (14.73)	88.89	Moderate to severe	275.29 days (176)	15 mg bd	28 days	Placebo	Cognition
Peattie et al. (40)	UK	RCT crossover	34	34.1 (10.7)	75	Mild to severe	21.3 months (11.9)	30 mg	Once	Placebo	Cognition
Plenger et al. (41)	USA	RCT parallel	23	28.69 (12.93)	73.91	Moderate to severe	NI	0.3 mg/kg bd	30 days	Placebo	Cognition Memory Motor function PCS
Speech et al. (42)	USA	RCT crossover	12	27.58 (5.59)	41.67	Moderate to severe	48.5 months (32.29)	0.3 mg/kg bd	1 week	Placebo	Cognition
Tiberti et al. (43)	Argentina	RCT parallel	10	38.6 (15.9)	60	NI	23.2 months (19.8)	10–40 mg (once/week)	4 weeks	Placebo	Cognition
Whyte et al. (44)	USA	RCT crossover	19	30.8 [17–75]	78.95	Moderate to severe	514 days [38–3,245]	0.25 mg/kg bd	3 days	Placebo	Cognition
Whyte et al. (9)	USA	RCT crossover	34	37 [20–55]	85	Moderate to severe	Median 3.2 years [4 months-34.2 years]	0.3 mg/kg bd	6 weeks	Placebo	Cognition
Willmott et al. (45)	Australia	RCT crossover	40	26.33 (9.14)	70	Mild to severe	68.38 days (77.09)	0.3 mg/kg bd	2 weeks	Placebo	Cognition
Willmott et al. (26)	Australia	RCT crossover (follow-up)	32	26.97 (9.37)	65.63	Moderate to severe	68 days, median 47 days	0.3 mg/kg bd	2 weeks	Placebo	Cognition
Zhang and Wang (10)	China	RCT parallel	36	35.6 (11.5)	75	Mild to moderate	44.8 days (7)	20 mg od	30 weeks	Placebo	Cognition Depression Fatigue

^*^GCS parameters: mild = 13–15; moderate = 9–12; severe = <8. Timing of GCS recording for definition of TBI severity varies across studies, as does stratification of TBI severity using a range of parameters (initial GCS, worst GCS, PTA duration).

Mean (standard deviation) [range]. MPh, methylphenidate; NI, no information; od, once daily administration; bd, twice daily administration; GCS, Glasgow Coma Scale; DaT, dopamine transporter (levels identified in the caudate region by fMRI).

### 3.3 Risk of bias analysis

**Figure 2:**
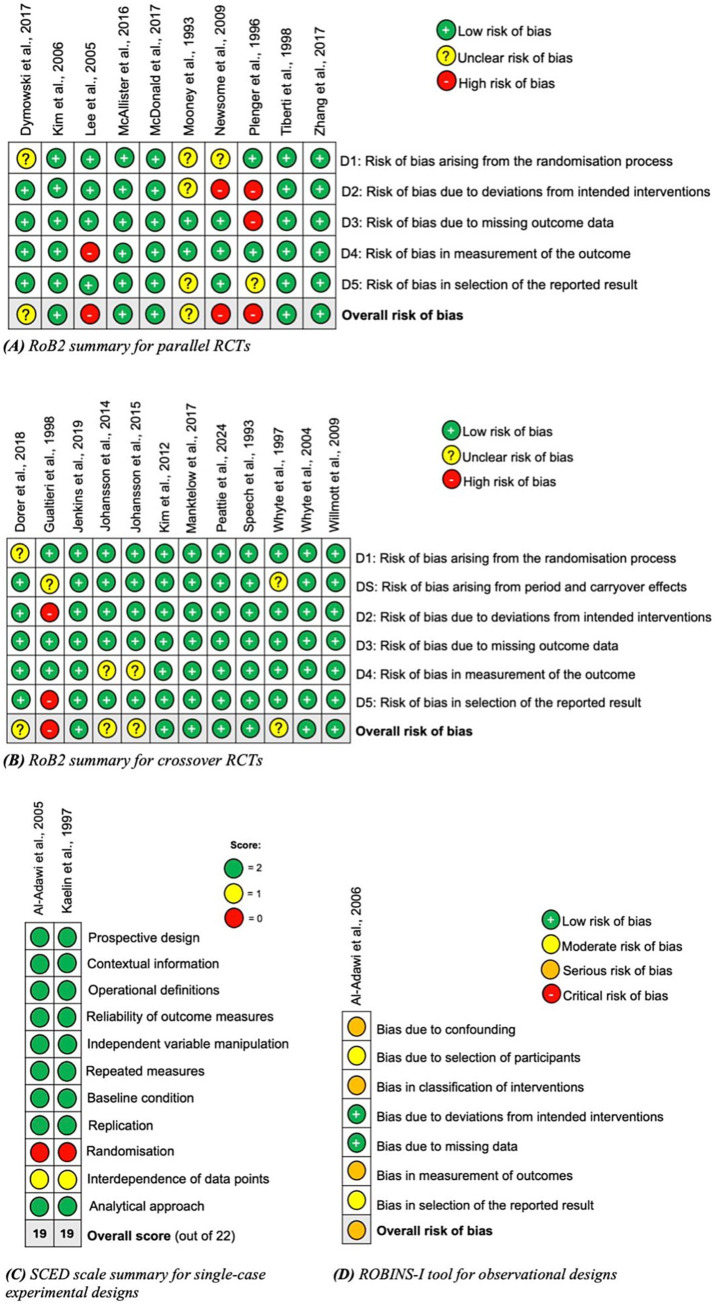
Risk of bias summaries using appropriate tools dependent on the study design: **(A)** parallel randomized controlled trials; **(B)** crossover randomized controlled trials; **(C)** single-case experimental designs; and **(D)** observational studies (45).

Risk of bias was assessed using the RoB2 tool, the SCED scale, and the ROBINS-I tool (18–20). Twelve studies were rated as low risk of bias, six as unclear, four as high and one as serious. Risk of bias summaries for each tool are shown in Figure 2, displayed using the SYRCLE tool format (46). Of the 10 parallel RCTs, the randomisation process and deviations from intended interventions were areas of common weakness. The crossover RCTs had a much lower risk of bias overall; period and carryover effects and outcome measurement domains were areas of weakness in these studies. The SCED studies rated poorly in the randomisation and independence of data point domains. The singular cohort study had serious risk of bias in the confounding, classification of interventions and measurement of outcomes domains.

### 3.4 Results for each outcome domain

#### 3.4.1 Aggression

Mooney et al. (38) directly addressed whether MPh use following TBI affects aggressive behaviour. This parallel RCT randomised 38 participants with severe TBI to receive MPh or placebo. Participants were mean 27 months post-injury (SD = 21.13 months). The State–Trait Anger Scale (STAS), Belligerence cluster score from the Katz Adjustment Scale and Anger-Hostility factor score of the Profile of Mood States were used as outcome measures. All outcome measures detected significant improvement following MPh administration in this cohort (*p* < 0.005), except the STAS state subsection (*p* = 0.06).

#### 3.4.2 Agitation

The systematic search identified no studies addressing the effect of MPh on agitation in TBI patients.

#### 3.4.3 Apathy

Jenkins et al. (30) measured the effect of MPh on apathy following TBI. Two distinct groups were identified on neuroimaging: low and normal dopamine transporter (DaT) levels in the caudate region of the brain. The Lille Apathy Rating Scale was implemented in both groups in a crossover RCT. The low caudate DaT group showed a significant reduction in both the self-completed and caregiver questionnaires following treatment with MPh (*p* = 0.03 and 0.02, respectively), equating to an improvement in apathy symptoms. No significant difference was noted in the normal caudate DaT group.

#### 3.4.4 Cognition

**Figure 3:**
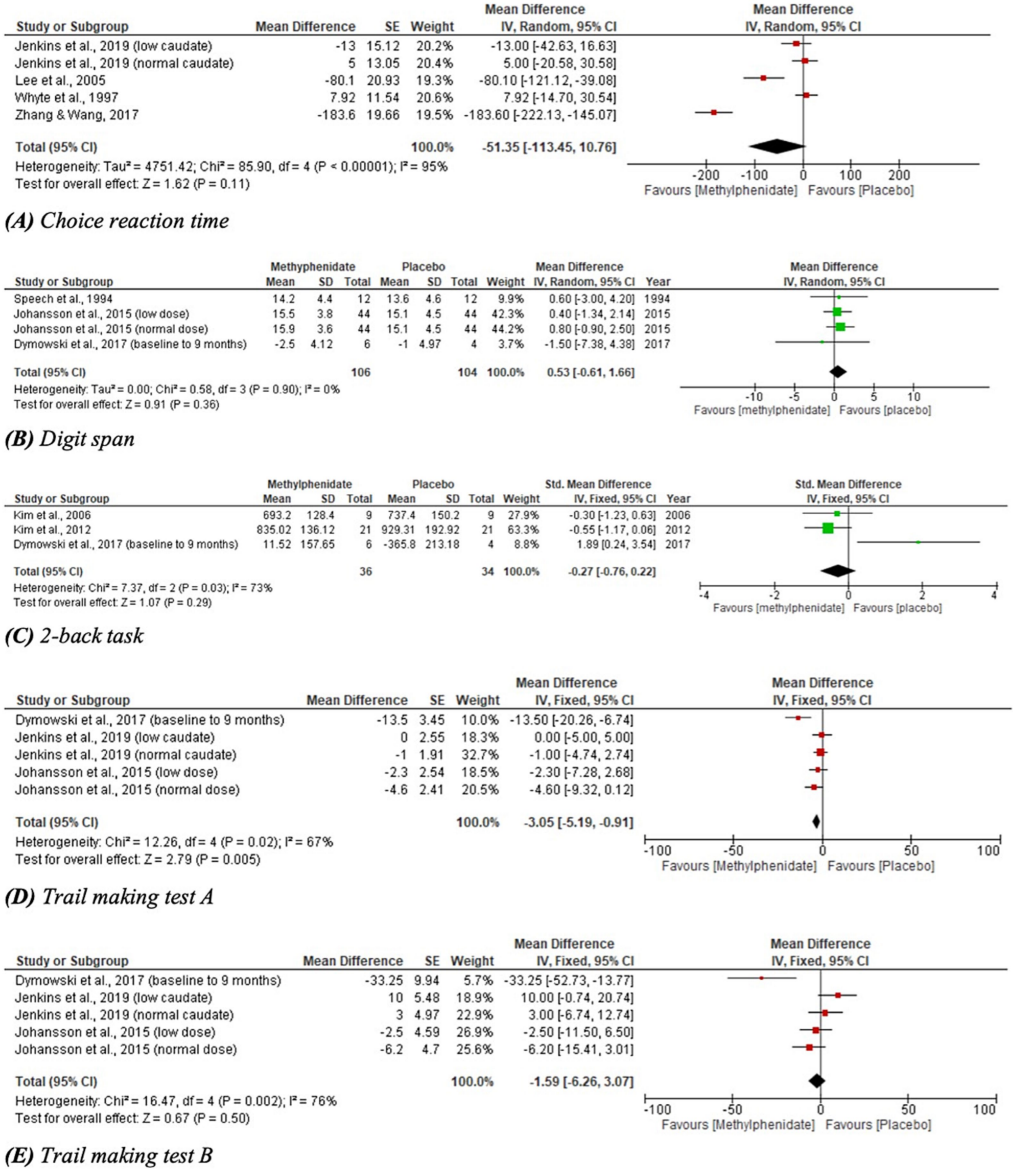
Meta-analysis of cognitive outcomes with MPh after TBI, based on varying outcome measures. SE, standard error; IV, inverse variance; CI, confidence interval; SD, standard deviation.

Twenty three studies measuring the effect of MPh on cognitive function were identified by the search, consisting of 628 participants. Sixteen studies recorded significant differences following MPh administration in one or more outcome measures. Two studies yielded indeterminable results (9, 35) and seven recorded no significant results regarding cognition (28, 29, 38–42). The results of this domain are presented in table format for clarity (Table 3). Four outcome measures for cognition were included in meta-analysis, shown in Figure 3 and analysed later in this section.

**Table 3:** Results for the cognition domain.

Study ID	Study design	*n*	Mean time since TBI (±SD) [range]	Outcome measures	Results
Al-Adawi et al. (27)	Single case experimental design (AAB)	10	68.08 days (46.24)	Useful Field of View tool (UFOV) Functional Independence Measure (FIM) cognitive subscale	Both cognitive outcome measures yielded statistically significant results (both UFOV and FIM *p* < 0.001 in all domains). Results of TBI participants were not reported independently of those with other brain injuries.
Al-Adawi et al. (28)	Prospective cohort	30	NI	Rancho Los Amigos levels of cognitive function tool FIM cognitive subscale	Neither outcome measure had statistically significant results (*p* = 0.479 and 0.411, respectively).
Dymowski et al. (29)	RCT parallel	11	293 days (104.76)	Rating Scale of Attentional Behaviour (RSAB) Simple and complex versions of the Selective Attention Task (SAT) Ruff 2&7 SAT (2&7) Trail Making Test (TMT) Digit Span (DS) Symbol Digit Modalities Test (SDMT)	No significant differences were identified in any outcome measure.
Gualtieri et al. (8)	RCT crossover	15	46.8 months (41.3)	Selective Reminding Test (SRT) 2&7 Verbal and Non-Verbal Fluency Tests (VFT and NVFT) TMT Continuous Performance Test Digit-Symbol Substitution Test (DSST) Benton Visual Retention Test	Significant results were found between baseline and high dose groups for the VFT (*p* = 0.017), and overall for the NVFT (*p* < 0.001). No other outcome measure yielded statistically significant results.
Jenkins et al. (30)	RCT crossover	40	Normal caudate DaT: 67 months (86) Low caudate DaT: 86 months (93)	Choice Reaction Time (CRT) TMT RSAB Cognitive Failures Questionnaire Delis-Kaplan Executive Function System (D-KEFS) People test for executive memory	The low caudate DaT group displayed significant change in CRT score following MPh administration (p = 0.02). No other outcome measure yielded significant results in either patient subset.
Johansson et al. (11)	RCT crossover	44	8.2 years (5.7)	TMT Digit Symbol Coding (DSC) DS A pre-specified computer test (simple and complex versions)	Significant differences were found in all cognitive domains between the no MPh condition and the normal dose (*p* = <0.007). No significant differences were found between no MPh and low dose, or low dose and normal dose. At 6 months follow-up, all cognitive tests reported significant changes from baseline (*p* < 0.001) except TMT D (*p* = 0.811). At mean 5.5 years follow-up, only DSC had significant results (*p* = 0.008)
Kaelin et al. (32)	Single case experimental design (AABA)	11	19.8 days (18.72)	Wechsler Adult Intelligent Scale-Revised (WAIS-R) DS subtest Wechsler Memory Scale-Revised (WMS-R) Mental Control subtest TMT Weschler Intelligence Scale for Children-III Symbol Search subtest Mesulam Verbal Cancellation Test	Significant improvement (*p* < 0.05) was seen following MPh administration for the DS, mental control and symbol search tests. Statistical tests could not be performed on the remaining outcome measures due to insufficient data availability.
Kim et al. (33)	RCT parallel	18	2.6 (1.95)	2-back working memory task Visuospatial Attention Task	The 2-back task produced significant result in participants’ reaction times (*p* < 0.05) but not in accuracy. The latter task produced no significant results.
Kim et al. (34)	RCT crossover	23	51.1 months (63.3)	2-back task Visual Sustained Attention Task	Participant accuracy and median reaction time were both significant for the former measure (*p* = 0.005 and < 0.05, respectively); the latter task yielded significant results for median reaction time only (*p* < 0.05).
Lee et al. (12)	RCT parallel	30	33.35 days (4.85)	CRT Critical Flicker Fusion Threshold Compensatory Tracking Task (CTT) Mental Arithmetic Test (MAT) Sternberg Memory Scanning Task (STM) DSST Mini-Mental State Examination (MMSE)	The recognition reaction time section of CRT yielded significant time effect results favouring MPh (*p* = 0.021). No other cognitive outcome measures recorded significant results.
Manktelow et al. (35)	RCT crossover	38	20.4 months (11.82)	N-back task using 0-, 1-, 2- and 3-back trials to achieve brain activation on fMRI N-back task with 0- and 2-back conditions during fMRI acquisition	Outcome measure scores were not reported, therefore impact of MPh on the outcome measures could not be ascertained.
McAllister et al. (36)	RCT parallel	32	NI	RNBI postmorbid cognitive scale Rey’s Auditory Visual Learning Test Digit Symbol TMT-A	At 12 weeks, the MPh group yielded significant results on the RNBI and digit symbol outcome measures compared to placebo (*p* = 0.036 and 0.011, respectively).
McDonald et al. (37)	RCT parallel	71	7.56 (9.27)	WAIS-III DS and DSC subtests D-KEFS Sorting Test Brown Location Test Wechsler Abbreviated Scale of Intelligence Vocabulary and Block Design subtests Craft Story Memory Test Grooved Pegboard Test Finger Tapping Test Thumb-finger Sequencing Test	The Brown location test yielded significant differences between MPh and placebo in the groups receiving MAAT (*p* = 0.021). There were no other significant results between MPh and placebo in either MAAT or ABT group.
Mooney et al. (38)	RCT parallel	38	27.08 months (21.13)	Letter Cancellation Test Variation of SRT	There was no significant difference between MPh and placebo in either outcome measure.
Newsome et al. (39)	RCT parallel	9	275.29 (176)	N-back task with 0- and 2-back conditions during fMRI acquisition	Neither MPh or placebo yielded significant results when comparing pre-treatment and post-treatment scores.
Peattie et al. (40)	RCT crossover	34	21.3 months (11.9)	Cambridge Neuropsychological Test Automated Battery (CANTAB)	No significant differences were detected.
Plenger et al. (41)	RCT parallel	23	NI	WMS-R memory and DS subtests 2&7 Disability Rating Scale (DRS) SRT Galveston Orientation and Amnesia Test Continuous Performance Test (Vigil) Paced Auditory Serial Addition Test (PASAT)	DRS yielded significant results at 30-days (p = 0.007), but not at 90 days (*p* = 0.12). This paper reported group outcome measure effects based on the category of cognition being measured. Tests for attention (including WMS-R subtests, 2&7, PASAT and continuous performance test) also yielded significant results at 30-days, but not at 90 days (*p* < 0.03 and 0.40, respectively). Similarly, the motor performance and memory domain, measured by the Porteus maze and pursuit rotor tests (p < 0.05 at 30-days and 0.07 at 90 days).
Speech et al. (42)	RCT crossover	12	48.5 months (32.29)	Gordon Diagnostic System WAIS-R DS and DSC subtests Stroop Interference Task Sternberg High Speed Scanning Task SRT Serial Digit Test	No outcome measured significant results in any domain.
Tiberti et al. (43)	RCT parallel	10	23.2 months (19.8)	WAIS WMS-R MMSE Buschke SRT DS TMT	No significant effects were recorded in any outcome measure.
Whyte et al. (44)	RCT crossover	19	514 days [38–3,245]	Sustained Arousal Task Phasic Arousal Task Distraction Task Behavioural Inattention Task CRT	MPh significantly improved reaction time and performance accuracy in the phasic arousal domain (*p* = 0.05 and 0.01, respectively). CRT also showed significant change in its slope of speed function, with a p value of 0.01.
Whyte et al. (9)	RCT crossover	34	Median 3.2 years [4 months-34.2 years]	CRT Sustained Arousal and Attention Tasks Speed/accuracy Trade-off Task Distraction Task Dual Task Sustained Attention to Response Task Inattentive Behaviour Task Classroom Attentiveness and Attention Ratings	Composite ratings of initial speed, caregiver ratings and on-task behaviour were reportedly improved with MPh, however *p* values were not reported so the significance of these results cannot be determined.
Willmott et al. (45)	RCT crossover	40	68.38 days (77.09)	2&7 SAT 4CRT SDMT Letter Number Sequencing Task Wechsler Test of Adult Reading (WTAR)	The speed measures yielded multiple significant scores; the 2&7 automatic measure (*p* = 0.003), the simple SAT reaction time measure (p = 0.001) and two measures of CRT (*p* = 0.002 and 0.017). None of the accuracy measures in any of the outcome measures yielded significant results.
Zhang et al. (10)	RCT parallel	36	44.8 days (7)	CRT CTT MAT DSST MMSE	All outcome measured yielded significant results (*p* < 0.001, <0.001, 0.02, <0.001 and < 0.001, respectively).

Meta-analyses for outcomes related to cognitive performance are given in Figure 3; one outcome measure yielded statistically significant results. CRT demonstrated a trend toward favouring MPh but did not reach statistical significance (*p* = 0.11, CI [−113.45, 10.76]) and considerable heterogeneity was identified (*I*^2^ = 95%). DS performance favoured placebo over MPh, but yielded no significant effect (*p* = 0.36, CI [−0.61, 1.66]). The 2-back task recorded a non-significant effect favouring MPh (*p* = 0.29, CI [−0.76, 0.22]) with substantial heterogeneity (*I*^2^ = 73%). TMT-A reported a statistically significant result favouring MPh (*p* = 0.005, CI [−5.19, −0.91]) with substantial heterogeneity (*I*^2^ = 67%). TMT-B demonstrated a trend toward favouring MPh but did not reach statistical significance (*p* = 0.50, CI [−6.26, 3.07]) and had substantial heterogeneity (*I*^2^ = 76%).

#### 3.4.5 Depression

**Figure 4:**
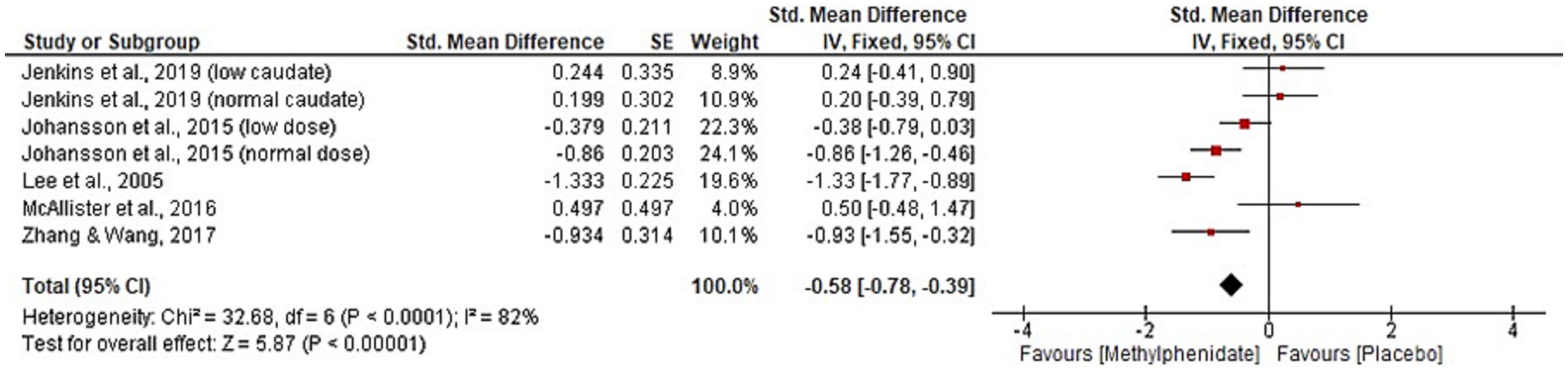
Meta-analysis of depression outcomes with MPh after TBI, based on varying outcome measures, synthesised using Cohen’s *d* to calculate standardised mean difference. SE, standard error; IV, inverse variance; CI, confidence intervals.

Six studies were identified reporting the effects of MPh on depression post-TBI, with a total of 217 participants. Whilst a range of outcome measures were used to monitor the effect of MPh on depression, it was possible to synthesise the standardised mean difference from multiple studies using Cohen’s *d* (23). Two parallel RCTs (10, 12) recorded depression using the Hamilton rating scale for depression (Ham-D) and Beck depression inventory (BDI). Zhang and colleagues (10) monitored 36 TBI patients (mean 46 days post-injury) randomised to receive MPh (up to 20 mg daily) or placebo. Lee and colleagues (12) randomised 30 participants (mean 35 days post-TBI) to MPh (up to 20 mg daily) or placebo arms. Both recorded significant improvement in Ham-D (*p* = 0.005 and < 0.001, respectively) and in BDI (*p* = 0.04 and < 0.05, respectively) following MPh. The Comprehensive Psychopathological Rating Scale (CPRS) also yielded significant results in two crossover RCTs. Johansson et al. (31) recorded significant improvement post-MPh (*p* = 0.001) in 24 participants (mean 8.6 years post-TBI) randomised to receive low dose MPh (15 mg daily), normal dose (20–60 mg daily) or placebo. Johansson and colleagues (11) randomised 44 participants (mean 8.2 years post-TBI) to three arms at the same doses as the previous study. This study and its follow-up papers (23, 24) recorded significant improvement following MPh and each dose (*p* < 0.001). The hospital anxiety and depression scale yielded no significant results (30), whereas the patient health questionnaire 9 showed significant improvement of MPh over placebo (*p* = 0.0009) (36). Meta-analysis for the depression domain is given in Figure 4. Johansson et al. (31) was excluded from meta-analysis due to insufficient data reporting. The findings are statistically significant (*p* < 0.00001, CI [−0.78, −0.39]) favouring MPh, with substantial heterogeneity among the included studies (*I*^2^ = 82%).

#### 3.4.6 Fatigue

**Figure 5:**
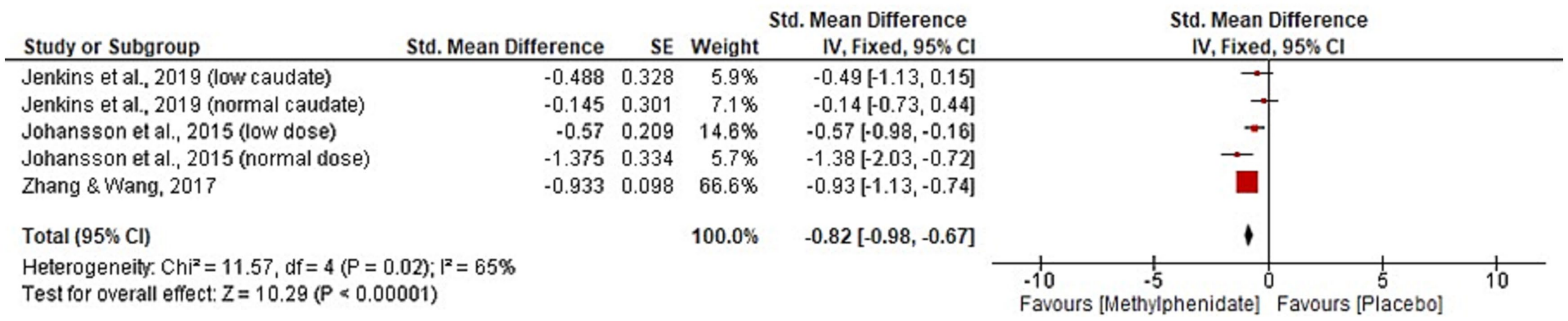
Meta-analysis of fatigue outcomes with MPh after TBI, based on varying outcome measures. Synthesised using Cohen’s *d* to calculate standardised mean difference. SE, standard error; IV, inverse variance; CI, confidence intervals.

Four studies, with a total of 144 participants, were identified that reported the effect of MPh on fatigue post-TBI. Three studies (10, 11, 30) reported the mental fatigue scale. Zhang and colleagues (10) found significant improvement post-MPh (*p* = 0.005) in a cohort of 36 TBI patients 46 days post-injury. Johansson et al. (11) reported significant improvement from MPh (*p* < 0.001) in 44 TBI patients (mean 8.2 years post-injury). This study implemented two follow-up papers at 6-months and 5.5 years post-baseline that reported continued significant improvement from MPh (*p* < 0.001) (23, 24). Johansson et al. (31) also reported significant results favouring MPh (*p* < 0.001) in its cohort of 24 participants (mean 8.6 years post-TBI). Jenkins and colleagues (30), that split participants based on caudate DaT levels, found significant improvement following MPh administration in both low and normal groups (*p* = 0.007 and 0.03, respectively) in the visual analogue scale of fatigue. Meta-analysis for the fatigue domain is displayed in Figure 5. One study was excluded from meta-analysis due to insufficient data reporting (31). The significant result favours MPh (*p* < 0.00001, CI [−0.98, −0.67]) and has substantial heterogeneity (*I*^2^ = 65%).

#### 3.4.7 Memory

Three studies included memory endpoints as secondary outcome measures (8, 12, 41), though memory was not the primary outcome of any study. Measuring verbal declarative memory using SRT, Gualtieri and colleagues (8) found no significant differences from baseline performance in TBI patients (47 months post-injury) after either low dose (0.15 mg/kg bd) or normal dose (0.30 mg/kg bd) MPh. Similarly, Plenger and colleagues (41) found that verbal declarative memory, measured using SRT, did not improve at 30- or 90-days post-MPh treatment (0.30 mg/kg daily). This study did identify short-term improvements (at 30 days) in procedural memory with MPh, though these were not observed at 90 days (*p* < 0.05 and 0.07, respectively). Lee and colleagues (12) implemented a parallel RCT to assess short-term memory using STM in TBI patients 35 days post-injury. Significant improvement was recorded in both MPh (up to 20 mg daily) and placebo groups (*p* < 0.01 and 0.001, respectively).

#### 3.4.8 Motor function

Two RCTs were identified that recorded motor function in 37 TBI patients receiving MPh. Dorer and colleagues (13) implemented a crossover design on 14 participants (23 months post-injury). Significant differences were detected on fMRI for reaction time between the MPh (30 mg) and placebo groups (*p* = 0.005). This was also demonstrated by Plenger and colleagues (41) in a parallel study design on 12 TBI patients (no information of time since injury given). At 30-days, a significant change in motor performance was observed (*p* < 0.05), but not at 90-days (*p* = 0.07).

#### 3.4.9 Post-concussion syndrome

Three parallel RCTs reported outcome measures for PCS, two of which recorded significant changes for MPh at various timepoints. The Rivermead post-concussive symptoms questionnaire was implemented in two parallel RCTs that both yielded significant results at 4 weeks. Lee and colleagues (12) monitored 30 participants (35 days post-TBI) for 4 weeks, detecting significant improvements in both MPh and placebo groups (*p* < 0.001 and < 0.005, respectively). MPh was administered at increasing intervals up to 20 mg daily. Similarly, McAllister and colleagues (36) randomised 32 participants to MPh (increasing dose up to 30 mg) or placebo. At 4 weeks, MPh was significantly superior to placebo (*p* = 0.0467); no significant results were detected at any other time-point. A symptom interview was conducted by Plenger and colleagues (41) on 32 participants randomised to receive MPh (0.3 mg/kg daily) or placebo for 30 days; no information was given on time since injury. No significant change was detected by this outcome measure.

#### 3.4.10 Sleep

Two studies looking at sleep were identified by the systematic search. Lee and colleagues (12) recorded multiple sleep domains of 30 participants (35 days post-TBI) who were randomised to receive MPh (up to 20 mg daily) or placebo. Four sleep domains (quality of sleep, awaking from sleep, behaviour following wakefulness and sleepiness) significantly improved with both MPh and placebo (*p* < 0.01 and < 0.05, respectively). Sleepiness and ease of getting to sleep were not significantly affected. A prospective cohort study (28) implemented regular surveying of participants (mean 76 days post-TBI) by staff who rated sleep patterns and apparent sleep state. Seventeen participants received MPh (5–10 mg daily). No significant effects were detected on daytime or night-time sleep patterns.

## 4 Discussion

This systematic review of 25 studies, including 666 participants, aimed to synthesise the current evidence base for the use of MPh to target cognitive and neuropsychological symptoms in the chronic sequelae of TBI. Due to significant heterogeneity in study design and outcome reporting, the opportunity for synthesis in meta-analyses was limited. Despite this, the available evidence suggests that MPh may improve depression, fatigue and elements of cognition following TBI. Five cognitive outcome measures were analysed, one of which yielded significant improvement following MPh administration. Depression and fatigue meta-analyses yielded significant improvement following intervention. The remaining neurobehavioural domains (aggression, agitation, apathy, memory, motor function, PCS and sleep disturbance) were not suitable for meta-analysis. Qualitative synthesis of these results yielded inconclusive results due to varied quality and low quantities of research into each area.

Existing meta-analyses have focused on specific neurobehavioural and cognitive domains rather than analysing the evidence on the use of a specific drug to address a range of domains. In the view of the authors, the principal attraction to MPh as a therapeutic agent in the rehabilitative stage after TBI is its potential to provide efficacy in improving symptoms across multiple domains. Furthermore, as many such domains are interlinked (e.g., depression, sleep and cognition), previous reviews which consider the benefits to discrete symptoms in isolation in our view are limited in evaluating the overall potential of MPh in TBI. This review collates all available evidence regarding MPh use in specified TBI sequelae and provides updated evidence synthesis on this topic, offering a comprehensive evaluation of its potential holistic benefits in TBI rehabilitation.

We found that there was significant inconsistency across studies regarding selection of outcome measures. A total of 70 distinct cognitive measures are reported across 23 studies; only the TMT-A yielded a significant result in meta-analysis, favouring MPh use. The TMT-A provides a reliable and valid assessment of an individual’s visual attention and speed of visual processing. Slowed processing speed has been found to be associated with poorer functional outcome (47), highlighting the importance of early identification and effective management within this population. The association between the administration of MPh and performance on the TMT-B (a test of visual attention and attentional switching) did not reach statistical significance. Despite this, attentional and executive dysfunction following a TBI is a strong predictor of poor functional outcome following a TBI (48), indicating the importance of successful treatment of these areas of cognition. The lack of association between MPh response and TMT-B performance may be due to the high heterogeneity of included studies, the variation in time following TBI in which cognitive assessment was conducted and psychological distress, therefore warranting further investigation into the reasons for these differences. Despite previous research citing significant association between mild TBI and ADHD (49), cognitive deficits associated with more severe TBI differ markedly to those of ADHD and as such, response to methylphenidate is likely to also considerably differ.

Previous meta-analyses of stimulant use post-TBI further support the use of MPh to enhance specific cognitive domains such as executive memory, attention and processing speed (50–52). However, no study supports the use of MPh for overall cognitive improvement in this cohort due to inconsistencies between studies and poor data availability. Risk of bias analysis yielded variable results. Of the nine studies included in cognition meta-analyses, three were rated as unclear risk of bias (11, 29, 44) and one as high (12), with common issues arising from measurement of outcome. This highlights the need for further studies with enhanced methodological rigour and consistent reporting of cognitive outcomes.

In the depression domain, this systematic review aligns with previous research in its support of MPh use (53, 54). Meta-analysis (Figure 4) found significant improvement in depressive symptoms, explained by the action of MPh on dopamine, a neurotransmitter involved in mood regulation (55, 56). MPh was reported as superior to sertraline at improving depressive symptoms in TBI patients (12), highlighting an area for future research to address. Like cognition, a range of outcome measures were reported; five outcome measures were used across six studies to report depression, and risk of bias was increased due to heterogeneity of outcome measurement. This emphasises the need for consistent outcome reporting.

The fatigue domain meta-analysis produced significant results supporting efficacy of MPh, concurring with previous research (57). As MPh is a stimulant, its action on alleviating fatigue, via its effects on alertness, motivation and perception of effort, is understandable (57). One study included in the meta-analysis had unclear risk of bias in the measurement of the outcome domain, potentially undermining our confidence in these findings.

Individual studies detected significant improvements in aggression, apathy, multiple cognitive domains, motor function, PCS and various sleep domains. However, the effect of MPh on these outcome domains identified by this review currently lacks sufficient quality and quantity of evidence to definitively support or reject the use of MPh post-TBI. The exclusion of studies conducted on patients under 72 h post-injury may be responsible for this evidence deficit. This time-restriction was placed on the systematic search due to the further heterogeneity that would be introduced by inclusion of patients with TBI in the acute stages of injury. The differences in mechanisms of action associated with instigating treatment in the acute stage of TBI would likely further skew results and hinder interpretation of MPh effectiveness (18). Additionally, each domain addressed in this review would interact differently with MPh at each stage of injury.

Complex biopsychosocial factors affect each domain included in this study and was not accounted for in any paper included in this review. Additionally, the existing research is limited by a diverse population sample, studies with small effect sizes and varied methodological rigor, indicating the need for higher quality primary studies conducted using standardised procedures, accounting for confounding factors. Should sufficient data be available, recruitment should focus on specific time points at which efficacy is theorised to be highest; the current data suggests increase effectiveness of MPh in the chronic stage of TBI (>3 months post-injury). Further to this, improved understanding of the pathophysiological and neurochemical mechanisms of long-term TBI sequelae, such as chronic inflammation, neurodegeneration and neurotransmitter dysregulation, and the complex interplay between neuropsychological sequelae of TBI would allow for more targeted hypotheses and enhance the reliability and interpretability of clinical trial results. To improve understanding of the potential benefits of MPh, thorough assessment across domains in future studies would be helpful in allowing sub-group analyses to determine the mechanism by which the effects of MPh are mediated after TBI. Future research should incorporate standardised scales and functional imaging modalities, such as fMRI, to map neuroplastic changes and allow accurate comparison of results between cohorts. Further investigation into the optimal timing, dose, and duration of MPh use following TBI is required, and adverse effects in this population should be closely monitored.

This review is limited by small samples sizes, inconsistent results reporting and inclusion of highly heterogenous patient samples, primarily in time since injury, injury severity and symptomatic burden. Additionally, the risk of publication bias, in which null results are less likely to be published, further limits this review as each domain, except cognition, yielded few results. All meta-analyses, except one (Digit Span, Figure 3B), were rated to contain considerable or substantial heterogeneity. This reduces the reliability of the results and necessitates sub-group analysis to prove the detected effects are generalisable across populations. Sub-group analysis was not conducted as few studies were identified for each meta-analysis, meaning sub-group analysis would further reduce sample size and reliability of results, risking overinterpretation of results and generation of misleading conclusions. Similarly, post-hoc exploratory analyses would not provide robust, reliable evidence in keeping with the systematic review protocol.

The study population is highly heterogenous in the severity of TBI and time elapsed since TBI. Both elements impact the effect and efficacy of pharmacotherapy at improving cognitive and neuropsychological deficits commonly seen post-TBI. Further to this, common TBI sequelae are strongly dependent on one another (47, 48, 50, 51), meaning marginal cognitive improvements may be a secondary effect of treatment of other neuropsychological sequelae. The side effect profile of MPh in the TBI population has not been explored in this paper, nor has it been extensively studied in the wider literature. Notably, existing research on effect of MPh on seizure activity in this population has yielded conflicting results (58, 59). Future research should aim to establish a risk–benefit ratio for the use of MPh in this population.

The use of Cohen’s *d* to calculate standardised mean difference allowed different outcome measures to be directly compared. However, differences between the unitless figures are context-dependent, and when combined with the considerable heterogeneity statistics, interpretation is challenging. The pooled effect sizes may not represent all studies included in the meta-analyses as studies with small sample sizes or high variance may skew results. It is not possible to ascertain whether the effects of MPh on any domain are time-dependent due to inconsistencies between included studies; as described above, the study populations included acute to chronic TBI patients with a range of severities, ages and injury mechanisms, limiting the generalisability of our results.

The heterogeneity in participant characteristics limits our ability to draw one-size-fits-all recommendations for the use of MPh following TBI. Future studies should stratify participants by injury severity and chronicity, using consistent post-injury stages, to refine treatment recommendations and improve the precision of MPh use in TBI rehabilitation. It is likely that specific subgroups with specific characteristics symptomatology are more likely to benefit from MPh after TBI: future large studies with well-defined subgroups would be helpful to delineate such characteristics. Moreover, MPh has known side effects and these were not addressed in this study since the primary objective was to assess the effect of MPh on a range of cognitive and neurobehavioural TBI sequelae, and not to conduct a full safety analysis and side effect profile. This was deemed out of scope for this study and has been adequately described elsewhere (60). Further to this, inconsistencies in MPh dosage and duration and patient characteristics in the available data would have limited our ability to conduct a rigorous analysis.

## 5 Conclusion

This study provides evidence supporting the efficacy of MPh to improve depression, fatigue and possibly elements of cognition following TBI, concurring with existing meta-analyses. A small number of studies with high heterogeneity were included in each meta-analysis, reducing the reliability of these conclusions. Significant results were identified by individual studies in multiple outcome domains, however heterogeneity between study populations, study design and outcome measures used limits the ability to state definitively the effect of MPh in these domains. This heterogeneity may be due to the variability in study populations, intervention protocols and outcome measurement tools and without further studies using more specific groups, and employing more consistent outcome measures, the results should be interpreted with caution. This review is the first to comprehensively synthesise all available literature on the use of MPh in adults following TBI. Further primary research with higher methodological rigor is required, followed by larger meta-analyses containing pre-specified sub-group analyses. MPh has multiple potential clinical applications in the adult TBI cohort, however further research in this field is required to definitively prove the effectiveness and safety of MPh in this population.

## Data Availability

The original contributions presented in the study are included in the article/Supplementary material, further inquiries can be directed to the corresponding authors.

## Footnotes

^1^ https://www.nice.org.uk/guidance/ng211

^2^ https://bnf.nice.org.uk/drugs/methylphenidate-hydrochloride/

^3^ www.training.cochrane.org/handbook

^4^ https://sites.google.com/site/riskofbiastool/welcome/rob-2-0-tool/current-version-of-rob-2

## Glossary

BDI: Beck Depression Inventory
CI: confidence interval
CPRS: Comprehensive Psychopathological Rating Scale
CRT: Choice Reaction Time
CTT: Compensatory Tracking Task
DaT: dopamine transporter
D-KEFS: Delis-Kaplan Executive Function System
DRS: Disability Rating Scale
DS: Digit Span
DSST: Digit-Symbol Substitution Test
FIM: Functional Independence Measure
Ham-D: Hamilton Rating Scale for Depression
IV: inverse variance
MAT: Mental Arithmetic Test
MMSE: Mini-Mental State Examination
MPh: methylphenidate
NVFT: Non-Verbal Fluency Test
PASAT: Paced Auditory Serial Addition Test
PCS: post-concussion syndrome
RSAB: Rating Scale of Attentional Behaviour
SAT: Selective Attention Task
SCED: single-case experimental design
SD: standard deviation
SDMT: Symbol Digit Modalities Test
SE: standard error
SRT: Selective Reminding Rest
STM: Sternberg Memory Scanning Task
TBI: traumatic brain injury
TMT: Trail Making Test
UFOV: Useful Field of Vision Tool
VFT: Verbal Fluency Test
WAIS (-R, -III): Wechsler Adult Intelligent Scale (-Revised, -3)
WMS-R: Wechsler Memory Test-Revised
2&7: Ruff 2&7 SAT
