# Supplementary material for: Methylphenidate for the cognitive and neurobehavioural sequelae of traumatic brain injury in adults: a systematic review and meta-analysis
Source: Front Neurol. 2025 Mar 5;16:1546080. doi: 10.3389/fneur.2025.1546080 (PMC11919662; doi:10.3389/fneur.2025.1546080)
Supplement: Supplementary file 1 [file Table_1.DOCX]

Supplementary Table 1: Values used for cognition meta-analyses.

|  | | **Intervention** | | | **Comparator** | | |
| --- | --- | --- | --- | --- | --- | --- | --- |
| **Outcome measure** | **Study ID (subgroup)** | **Mean** | **SD** | **n** | **Mean** | **SD** | **n** |
| DS | Dymowski et al., 2017 (baseline to 9 months) | 30.25 | 4.93 | 6 | 24.25 | 7.37 | 4 |
|  | Johansson et al., 2015 (low dose) | 15.5 | 3.8 | 44 | 15.1 | 4.5 | 44 |
|  | Johansson et al., 2015 (normal dose) | 15.9 | 3.6 | 44 | 15.1 | 4.5 | 44 |
|  | Speech et al., 1994 | 14.2 | 4.4 | 12 | 13.6 | 4.6 | 12 |
| N-back 2-back | Dymowski et al., 2017 (baseline to 9 months) | 1027.44 | 194.89 | 6 | 957.31 | 261.91 | 4 |
|  | Kim et al., 2006 | 693.2 | 128.4 | 9 | 737.4 | 150.2 | 9 |
|  | Kim et al., 2012 | 835.02 | 136.12 | 21 | 929.31 | 192.92 | 21 |

Supplementary Table 2: Values used for cognition meta-analyses.

|  | | **Intervention** | | |
| --- | --- | --- | --- | --- |
| **Outcome measure** | **Study ID (subgroup)** | **Mean difference** | **Standard error** | **n** |
| TMT-A | Dymowski et al., 2017 (baseline to 9 months) | -13.5 | 3.45 | 6 |
|  | Jenkins et al., 2019 (low caudate) | 0 | 2.55 | 18 |
|  | Jenkins et al., 2019 (normal caudate) | -1 | 1.91 | 22 |
|  | Johansson et al., 2015 (low dose) | -2.3 | 2.54 | 44 |
|  | Johansson et al., 2015 (normal dose) | -4.6 | 2.41 | 44 |
| TMT-B | Dymowski et al., 2017 (baseline to 9 months) | -33.25 | 9.94 | 6 |
|  | Jenkins et al., 2019 (low caudate) | 10 | 5.48 | 18 |
|  | Jenkins et al., 2019 (normal caudate) | 3 | 4.97 | 22 |
|  | Johansson et al., 2015 (low dose) | -2.5 | 4.59 | 44 |
|  | Johansson et al., 2015 (normal dose) | -6.2 | 4.7 | 44 |
| CRT | Jenkins et al., 2019 (low caudate) | -13 | 15.12 | 20 |
|  | Jenkins et al., 2019 (normal caudate) | 5 | 13.05 | 20 |
|  | Lee et al., 2005 | -80.1 | 20.93 | 30 |
|  | Whyte et al., 1997 | 7.92 | 11.54 | 19 |
|  | Zhang & Wang, 2017 | -183.6 | 19.66 | 36 |

Supplementary Table 3: Values used for depression and fatigue meta-analyses.

|  | | **Intervention** | | |
| --- | --- | --- | --- | --- |
| **Outcome domain** | **Study ID (subgroup)** | **Cohen’s d** | **Standard error d** | **n** |
| Depression | Jenkins et al., 2019 (HADS depression, low caudate) | 0.244 | 0.335 | 18 |
|  | Jenkins et al., 2019 (HADS depression, normal caudate) | 0.199 | 0.302 | 22 |
|  | Johansson et al., 2015 (CPRS depression, low dose) | -0.379 | 0.211 | 44 |
|  | Johansson et al., 2015 (CPRS depression, normal dose) | -0.86 | 0.203 | 44 |
|  | Lee et al., 2005 (Ham-D) | -1.333 | 0.225 | 10 |
|  | Lee et al., 2005 (BDI) | -0.334 | 0.097 | 10 |
|  | McAllister et al., 2016 (PHQ9, week 12) | 0.497 | 0.497 | 32 |
|  | Zhang & Wang, 2017 (Ham-D) | -0.934 | 0.314 | 18 |
|  | Zhang & Wang, 2017 (BDI) | -0.674 | 0.323 | 18 |
| Fatigue | Jenkins et al., 2019 (VAS-F, low caudate) | -0.488 | 0.328 | 18 |
|  | Jenkins et al., 2019 (VAS-F, normal caudate) | -0.145 | 0.301 | 22 |
|  | Johansson et al., 2015 (MFS, low dose) | -0.57 | 0.209 | 44 |
|  | Johansson et al., 2015 (MFS, normal dose) | -1.375 | 0.334 | 44 |
|  | Zhang & Wang, 2017 (MFS) | -0.933 | 0.098 | 18 |
